# Risk of second cancers after the diagnosis of Merkel cell carcinoma in Scandinavia

**DOI:** 10.1038/sj.bjc.6605989

**Published:** 2010-11-16

**Authors:** D Bzhalava, F Bray, H Storm, J Dillner

**Affiliations:** 1 Division of Medical Microbiology, Department of Laboratory Medicine, Malmö University Hospital, Lund University, Entrance 78, Malmö SE-20502, Sweden; 2 Department of Laboratory Medicine, Karolinska Institute, Stockholm, Sweden; 3 Cancer Registry of Norway,Oslo, Norway; 4 International Agency for Research on Cancer, Lyon, France; 5 Department Cancer Prevention and Documentation, Danish Cancer Society, Copenhagen, Denmark; 6 Department of Medical Epidemiology & Biostatistics, Karolinska Institute, Stockholm, Sweden

**Keywords:** incidence, merkel cell carcinoma, merkel cell polyomavirus, second cancer

## Abstract

**Background:**

Merkel cell carcinoma (MCC) is an aggressive neuroendocrine tumour of the skin that has been associated with a new tumour virus, the MCC polyomavirus.

**Methods:**

To investigate whether MCC may have a shared aetiology with other cancers, we investigated the risk of second cancers after the diagnosis of MCC using the national cancer registries in Denmark, Norway and Sweden.

**Results:**

The overall cancer incidence was increased among patients diagnosed with MCC compared with the general population in these countries (79 secondary cancers total, Standardized Incidence Ratio (SIR) 1.38 (95% confidence interval (CI): 1.10–1.72); 49 secondary cancer in females, SIR 1.7 (95% CI: 1.29–2.25); 30 secondary cancers in males and SIR 1.05 (95% CI: 0.73-1.5)). There were significantly increased incidence ratios for non-melanoma skin cancers (34 secondary cancers, SIR 8.35 (95% CI: 5.97–11.68)), melanoma of skin (6 secondary cancers, SIR 4.29 (95% CI: 1.93–9.56)) and laryngeal cancer (2 secondary cancers, SIR 9.51 (95% CI: 2.38–38)). The SIRs for these three cancer sites were also elevated on restricting the follow-up to cancers occurring at least one year after MCC diagnosis.

**Conclusions:**

Patients diagnosed with MCC are at increased risk of a second cancer, particularly, other skin cancers. Conceivable explanations include the impact of increased surveillance of the skin and shared causative factors, for example, ultraviolet light exposure or MCC polyomavirus infection.

Merkel cell carcinoma (MCC) is a rare and aggressive neuroendocrine cutaneous tumour of the skin (Howard *et al*, 2006; Liao 2008). Incidence rates for MCC are age dependent and increase dramatically after the age of 50 years (Hussain *et al*, 2010). Merkel cell carcinoma harbours a newly discovered tumour virus, the MCC polyomavirus (MCV) (Feng *et al*, 2008) in the majority of cases (Feng *et al*, 2008; Kassem *et al*, 2008; Duncavage *et al*, 2009). MCC polyomavirus is clonally integrated in MCC tumours (Duncavage *et al*, 2009).

The epidemiology of MCV infection is as yet not known, and bidirectional evaluation of whether patients with MCV-associated disease are at excess risk of other diseases may provide important clues that generate hypotheses for further study. Howard *et al*, 2006 evaluated the reciprocal risks of multiple primary cancers among patients with MCC and significant associations were reported for cancers of the salivary gland, brain and other organs and unspecified parts of biliary tract, as well as multiple myeloma, chronic lymphocytic leukaemia and NHL (Howard *et al*, 2006). The Finnish Cancer Registry reported significantly increased risks for basal cell carcinoma of the skin and chronic lymphocytic leukaemia after the diagnosis of MCC (Koljonen *et al*, 2010).

Associations may in part be explained by aetiological factors shared between MCC and other tumour types and MCV infection could be one of these factors (Koljonen *et al*, 2010). However, to avoid epidemiological biases, such as selection bias or chance findings, large studies based on quality-assured and comparable data sets from population-based cancer registries are preferable.

We used the nationwide Danish, Norwegian and Swedish Cancer Registries to investigate the risk of developing second primary cancer after the diagnosis of MCC. The data from these Registries are based on mandatory notification and multiple sources and have been evaluated as accurate and close-to-complete (Mattsson *et al*, 1984; Tulinius *et al*, 1992; Storm *et al*, 1997; Larsen *et al*, 2009). In addition, the vast majority of cancers registered are based on histological diagnosis, creating a reliable basis for assessment of the risk of second cancers following the diagnosis of MCC (Koljonen *et al*, 2010).

## Materials and methods

Population-based national cancer registries were established in the Nordic countries in the 1940 and 1950s, and are considered to have a consistently high degree of comparability and completeness over time (Mattsson *et al*, 1984; Tulinius *et al*, 1992; Storm *et al*, 1997; Larsen *et al*, 2009). Patients with a first primary cutaneous MCC and other first primary cancer were identified from Danish, Norwegian and Swedish population-based cancer registries.

The study cohort includes all persons who had been registered with MCC over the calendar period of 1980–2007 in Denmark and during 1990–2007 in Norway and Sweden. The follow-up started on the date of the diagnosis of MCC and ended on the date of death, emigration or the closing date of the study (31 December 2007), whichever occurred first. Cancers occurring within 6 months of MCC diagnosis, as well as secondary MCC following primary MCC, were excluded from the analysis.

The observed subsequent primary cancers, occurring at least 6 months or at least 1 year after MCC diagnosis, were compared with those expected by incidence rates among the national populations. To estimate the expected number of cancers, the age-, sex- period- and site-specific incidence rates (extracted from NORDCAN) were multiplied by the respective numbers of accumulated person-years at risk. The ratios of the observed-to-expected number of cases were expressed as the standardized incidence ratio (SIR). Risks were also estimated stratified by the time that had passed since diagnosis of MCC (6–12 months, 1–4 years and ⩾5 years). 95% confidence intervals (CIs) of the SIRs were computed assuming a Poisson distribution for observed cases. Findings were considered significant for a two-tailed test with significance level *P*<0.05.

## Results

A total of 756 individuals were diagnosed with MCC, of which 716 had MCC as a first cancer (Table 1). Four subjects had MCCs detected simultaneously with another cancers and 36 subjects had MCCs after another cancer. None of these 40 patients had any additional cancers after 6 months since MCC diagnosis. All MCC diagnoses were based on histological examination. In total 23 of them were diagnosed before the year 1992. Only 10 patients were younger than 45 years at the time of the diagnosis, whereas 530 were 75 years of age or older. More patients were female (*n*=442, 60%), resulting in a female to male ratio of 1.4 : 1. The mean follow-up time after the diagnosis of MCC was 3.5 years. The cumulative post-diagnosis follow-up time yielded 2632.5 person-years at risk (Table 1).

Following a diagnosis of MCC, 142 second primary cancers were diagnosed in the study cohort. In total 79 and 65 second primaries were diagnosed after 6 months and 1 year, respectively, since MCC diagnosis. The most frequent second primary cancer was non-melanoma of the skin followed by melanoma of skin, breast cancer, lung cancer and rectal cancer (Tables 2 and 3).

Patients diagnosed with MCC had a greater risk for a second primary cancer than expected in a gender-, age- and calendar period-matched general population. The overall SIR for any subsequent cancer at any site, occurring at least after 6 months, was 1.38 (95% CI: 1.1–1.72). Female MCC patients were diagnosed with 49 subsequent cancers (SIR 1.7 (95% CI: 1.29–2.25)) and male patients with 30 cancers (SIR 1.05 (95% CI: 0.73–1.5)).

The most frequent second primary cancer was non-melanoma skin cancer (*n*=34; SIR 8.35 (95% CI: 5.97–11.68)). The risks of melanoma of skin (*n*=6; SIR 4.29 (95% CI: 1.93–9.56)) and laryngeal cancers (*n*=2; SIR 9.51 (95% CI: 2.38–38)) were also significantly increased (Table 2). These risks were significantly increased also in analyses restricted to cases occurring greater than 1 year after MCC diagnosis (Table 3). Stratification of follow up time did not markedly affect the overall risk of second primary cancers (Table 4).

## Discussion

We found that MCC patients were at increased risk of second primary cancers, in particular for non-melanoma and melanoma skin cancers. There was also a significantly increased risk for laryngeal cancers, but this was based on small numbers. Previously two registry-based studies have investigated the cancer risks among patients diagnosed with MCC (Howard *et al*, 2006; Koljonen *et al*, 2010). In the SEER program in the United States (Howard *et al*, 2006), the risk of overall second malignancy after MCC diagnosis was 1.2 (95% CI: 1.0–1.5). There were also significantly elevated SIRs for salivary gland tumours, biliary tract cancer and non-Hodgkin lymphoma. In the Finnish Cancer Registry (Koljonen *et al*, 2010), the SIRs for cancers at any site after MCC diagnosis was 2.3 (95% CI: 1.6–3.3). The SIRs of basal cell carcinoma and chronic lymphocytic leukaemia showed significantly increased risk among MCC patients (Koljonen *et al*, 2010). Selection/surveillance biases, different qualities of different cancer registries, rare cancers and/or unstable findings are conceivable explanations for the fact that the three different studies have failed to identify the same cancer forms as the most frequently occurring secondary primaries following MCC diagnoses.

The small number of patients with other cancers occurring before or on the same date as MCC preclude a more detailed analysis of whether any cancer forms are linked also in the other direction, that is, whether MCC risks are increased after other cancer. However, it is interesting to note that not less than 26 out of the 40 other cancers occurring before or on the same date as MCC were non-melanoma skin cancers, suggesting that the link between MCC and non-melanoma skin cancer may be bidirectional.

Shared aetiological factors which may conceivably explain the increased risk of a second primary cancer after the diagnosis of MCC include exposure to ultraviolet-radiation, immunosuppression or MCV infection. Exposure to sunlight may be an explanation of concomitant associations of MCC, non-melanoma skin cancer and melanoma. Also, MCV DNA is reported to be frequently found in the skin, not only in MCC tissue (Feng *et al*, 2008; Becker *et al*, 2009), and involvement of MCV infection also in other skin diseases is, therefore, plausible.

Surveillance bias after diagnosis should always be considered as an explanation for excess risk of secondary cancers. Analysis of trends of excess risks over time after diagnosis is conventionally used to search for surveillance bias, as medical attention is intensified immediately after a cancer diagnosis. We excluded cases occurring less than 6 months after MCC diagnosis in our analysis and we also found limited changes in estimates by restricting the study to cancers occurring greater than 1 year after diagnosis. Thus, although we have no evidence for surveillance bias, it still should be considered as a likely explanation of our findings. Especially for skin cancers, there may be a prolonged increased vigilance, not only from physicians but also from the patients themselves. The fact that the excess risk of skin cancer was essentially restricted to females is difficult to explain, but it is conceivable that gender-related differences in skin surveillance may exist.

The assessment of the risk for second cancers requires a large study cohort and a long study time. MCC is a rare and aggressive cancer associated with a poor prognosis. As it occurs mostly in elderly patients, the numbers of person-years available for follow-up is limited. Although we combined data from three countries, a somewhat inadequate number of person-years of follow-up is still a limitation of our study.

The major strength of our study is that the data are from cancer registries of three Nordic countries. These population-based cancer registries provide long-term national data that are considered to be of comparably high quality, with the majority of diagnoses among cases histologically confirmed (Tulinius *et al*, 1992).

We conclude that the incidence of second primary cancers is increased among patients diagnosed with MCC compared with the general population. Patients diagnosed with MCC are at an excess risk for malignant melanoma, larynx cancer and, in particular, for non-melanoma skin cancer.

## Figures and Tables

**Table 1:** Numbers of patients diagnosed with MCC and the person-years at risk for a second cancer stratified by age and sex

	**Number of subjects diagnosed with MCC**			
**Age at diagnosis**	**Males**	**Females**	**Total**	**Person-years at risk following the MCC diagnosis**
<29	1	1	2	25.6
30–44	2	6	8	74.3
45–59	18	17	35	157
60–74	83	98	181	862.4
>75	210	320	530	1513
Total	314	442	756	2632.5

Abbreviation: MCC=merkel cell carcinoma.

**Table 2:** Secondary cancers occurring at least 6 months after an MCC diagnosis

**Cancer site**	**Observed**	**Expected**	**SIR**	**(95% CI)**
All types	79	57.4	1.4	(1.1–1.72)
Non-melanoma of the skin	34	4.07	8.4	(5.97–11.68)
Female breast	6	4.91	1.2	(0.55–2.72)
Colorectal	6	7.88	0.8	(0.34–1.7)
Melanoma of skin	6	1.4	4.3	(1.93–9.56)
Lung	5	4.13	1.2	(0.5–2.91)
Prostate	3	7.72	0.4	(0.31–1.2)
Bladder etc.	3	3.19	0.9	(0.3–2.92)
Leukaemia	3	1.15	2.6	(0.84-8.09)
Larynx	2	0.21	9.5	(2.38–38.04)
Brain central nervous system	1	0.84	1.2	(0.17–8.44)
Kidney	1	0.91	1.1	(0.15–7.79)
Liver	1	0.52	1.9	(0.27–13.74)
Non-Hodgkin lymphoma	1	1.43	0.7	(0.1–4.97)
Other female genital organs	1	0.34	2.9	(0.41–20.9)
Ovary etc.	1	1.46	0.7	(0.1–4.87)

Abbreviations: CI=confidence interval; MCC=merkel cell carcinoma; SIR=standardized incidence ratio.

**Table 3:** Secondary cancers occurring at least 1 year after a MCC diagnosis

**Cancer site**	**Observed**	**Expected**	**SIR**	**(95% CI)**
All types	65	57.4	1.13	(0.89–1.44)
Non-melanoma of the skin	27	4.07	6.63	(4.55–9.67)
Female breast	5	4.91	1.02	(0.42–2.45)
Colorectal	5	7.88	0.63	(0.26–1.53)
Lung	5	4.13	1.21	(0.5–2.91)
Melanoma of skin	5	1.4	3.58	(1.49–8.6)
Prostate	3	7.72	0.39	(0.13–1.2)
Bladder etc.	3	3.19	0.94	(0.3–2.92)
Larynx	2	0.21	9.51	(2.38–38.04)
Brain central nervous system	1	0.84	1.19	(0.17–8.44)
Kidney	1	0.91	1.1	(0.15–7.79)
Liver	1	0.52	1.94	(0.27–13.74)
Leukaemia	1	1.15	0.87	(0.12–6.17)
Non-Hodgkin lymphoma	1	1.43	0.7	(0.1–4.97)
Other female genital organs	1	0.34	2.94	(0.41–20.9)
Ovary etc.	1	1.46	0.69	(0.1–4.87)

Abbreviations: CI=confidence interval; MCC=merkel cell carcinoma.

**Table 4:** Overall secondary cancers stratified by follow-up

**Follow-up**	**Observed**	**Expected**	**SIR**	**(95% CI)**
6–12 months	14	6.08	2.3	(1.36–3.89)
1–4 years	41	9.14	4.49	(3.3–6.09)
⩾5 years	24	8.91	2.69	(1.8–4.02)

Abbreviations: CI=confidence interval; SIR=standardized incidence ratio.
